# Non-Enzymatic Glucose Biosensor Based on CuO-Decorated CeO_2_ Nanoparticles

**DOI:** 10.3390/nano6090159

**Published:** 2016-08-26

**Authors:** Panpan Guan, Yongjian Li, Jie Zhang, Wei Li

**Affiliations:** Department of Materials Science, Taiyuan University of Technology, Taiyuan 030024, China; ddwhlw1980@sohu.com (P.G.); leeyj2015@163.com (Y.L.); sunxuejia@tyut.edu.cn (J.Z.)

**Keywords:** cerium oxide, copper oxide, biosensor, glucose, nanocomposite

## Abstract

Copper oxide (CuO)-decorated cerium oxide (CeO_2_) nanoparticles were synthesized and used to detect glucose non-enzymatically. The morphological characteristics and structure of the nanoparticles were characterized through transmission electron microscopy, X-ray photoelectron spectroscopy, and X-ray diffraction. The sensor responses of electrodes to glucose were investigated via an electrochemical method. The CuO/CeO_2_ nanocomposite exhibited a reasonably good sensitivity of 2.77 μA mM^−1^cm^−2^, an estimated detection limit of 10 μA, and a good anti-interference ability. The sensor was also fairly stable under ambient conditions.

## 1. Introduction

Glucose biosensors have been extensively investigated because of their promising applications in clinical chemistry, the food industry, and environmental protection [1,2,3]. In general, an enzyme is used as a major sensitive element in a glucose biosensor to detect glucose. Despite a good sensing performance, an enzyme-based glucose biosensor is usually limited by unstable glucose oxidase, a complex immobilization process, and high enzyme costs [4,5]. To overcome these limitations, researchers developed non-enzymatic glucose biosensors. Without enzymes, non-enzymatic glucose biosensors require a suitable sensing material, which can catalyze redox reactions and efficiently transfer electrons to electrodes [6]. Nanocomposites are promising and suitable sensing materials because the combination of components can provide an extended interface through which charge and energy transport are significantly enhanced [7].

Cerium oxide (CeO_2_) is a potential material for enzymatic glucose biosensors because of its unique properties, such as chemical inertness, biocompatibility, nontoxicity, and its ability to be suitable isoelectric point for enzyme adsorption [8]. CeO_2_ can highly induce electron transfer; as such, this material is a very suitable electron conductor for enzymatic biosensors. However, CeO_2_ is seldom used for non-enzymatic glucose biosensors because CeO_2_ fails to exhibit specificity and thus catalyze a surface redox reaction [6,9]. Copper oxide (CuO) has outstanding electrocatalytic activity [4]. The combination of CuO with CeO_2_ may maximize the advantages of these two materials and promote the application of CeO_2_ to non-enzymatic biosensors. Since finding a suitable sensing material is a primary task in research focusing on non-enzymatic biosensors, a CuO/CeO_2_ nanocomposite was prepared in this work and used for the first time as a non-enzymatic glucose biosensor.

## 2. Results and Discussion

**Figure 1:**
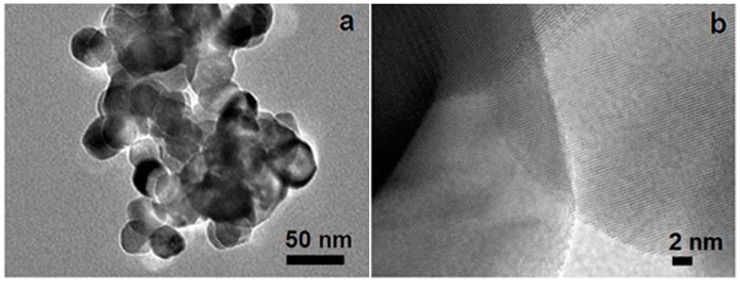
(**a**) Transmission electron microscopy (TEM) and (**b**) high-resolution transmission electron microscopy (HRTEM) image of copper oxide (CuO)/cerium oxide (CeO_2_) nanocomposite.

A typical transmission electron microscopy (TEM) image of a CuO/CeO_2_ nanocomposite is shown in Figure 1a. The sizes of the nanoparticles are approximately 20–50 nm. A high-resolution TEM image reveals randomly oriented nanocrystals with very clear lattice fringes (Figure 1b). All of these nanocrystals yield lattice distances of 0.31 ± 0.01 nm, which correspond to the (111) plane of the CeO_2_ crystal phase [10].

**Figure 2:**
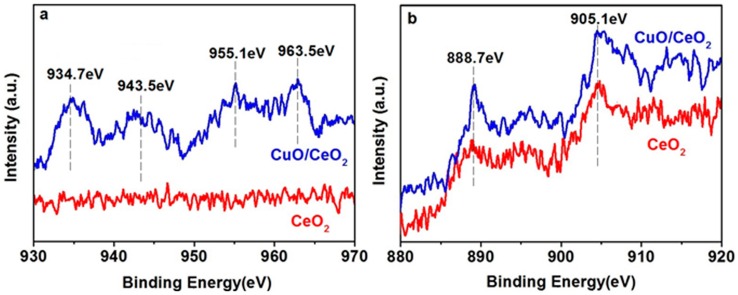
X-ray photoelectron spectroscopy (XPS) spectra of (**a**) Cu 2p and (**b**) Ce 3d.

Although no CuO particles are found in the TEM image, their existence can be evidenced by X-ray photoelectron spectroscopy (XPS), a powerful tool that examines material composition. The XPS spectra of Cu 2p are shown in Figure 2a. In comparison with the spectrum of the pure CeO_2_ sample, the spectrum of the nanocomposite reveals four peaks. The peaks at 934.7 eV and 955.1 eV are related to Cu 2p_3/2_ and Cu 2p_1/2_ peaks, respectively [11]. The peaks at 943.5 eV and 963.5 eV are assigned to the shake-up satellite peaks, which indicate the formation of CuO [4]. The XPS spectra of Ce 3d are shown in Figure 2b. The two evident peaks at approximately 888.7 and 905.1 eV correspond to the Ce^4+^ oxidation state in CeO_2_ [11,12].

**Figure 3:**
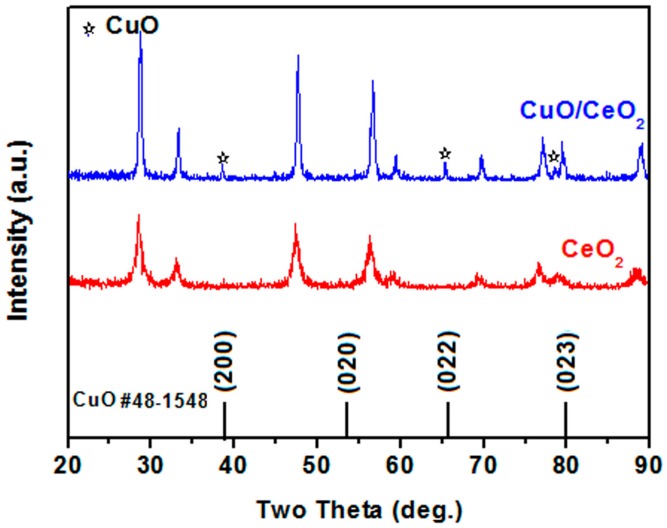
X-ray diffraction (XRD) patterns of CeO_2_ and CuO/CeO_2_ nanocomposite.

The X-ray diffraction (XRD) patterns of the CeO_2_ and CuO/CeO_2_ nanocomposites are shown in Figure 3. The peaks at 28.5°, 33.3°, 47.6°, 56.5°, 58.6°, 69.1°, 76.1°, 78.6°, and 88.3° correspond to the (111), (200), (220), (311), (222), (400), (331), (420), and (422) planes of the CeO_2_ crystal phase, respectively [8]. The appearance of these peaks implies that CeO_2_ exhibits a cubic fluorite structure. Three additional peaks at 38.5°, 65.1°, and 78.3° in the CuO/CeO_2_ nanocomposite pattern correspond to the (111), (022), and (023) planes of the monoclinic CuO [13]. Furthermore, these three peaks likely shift toward the lower 2θ positions than those in the JCPDS card data, such as Nos. 48–1548 and 80–1917, possibly because the interfacial Ce atom with a larger atomic radius causes the lattice strain of CuO.

**Figure 4:**
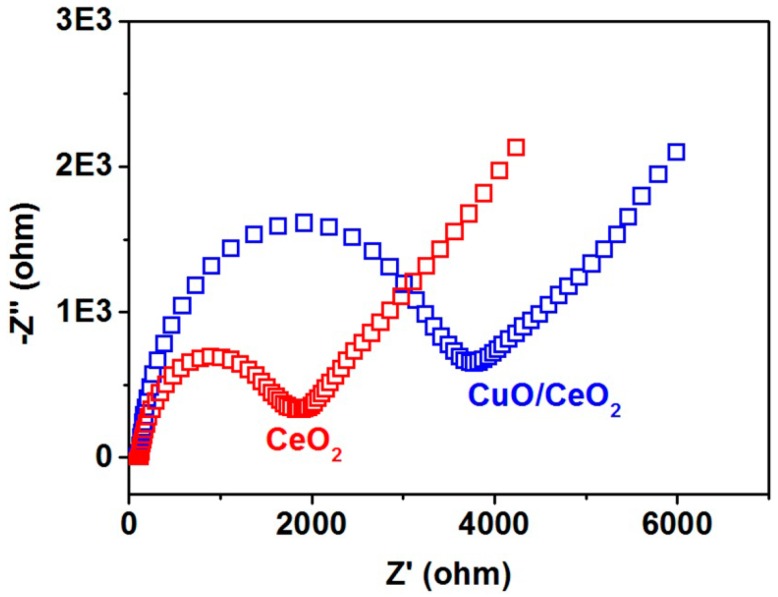
Nyquist impedance plots for CeO_2_/indium-tin oxide (ITO) and CuO/CeO_2_/ITO electrodes recorded in 0.1 M KCl solution containing 1 mM Fe[(CN)_6_]^3^^−^^/4−^ (1:1).

Figure 4 shows the electrochemical impedance spectroscopy of electrodes. The semicircular radius of the CuO/CeO_2_ nanocomposite is higher than that of pure CeO_2_. This finding indicates that the CuO/CeO_2_ nanocomposite yields a higher magnitude of charge transfer resistance than pure CeO_2_ does. CeO_2_ exhibits a higher electron transfer ability than CuO does.

**Figure 5:**
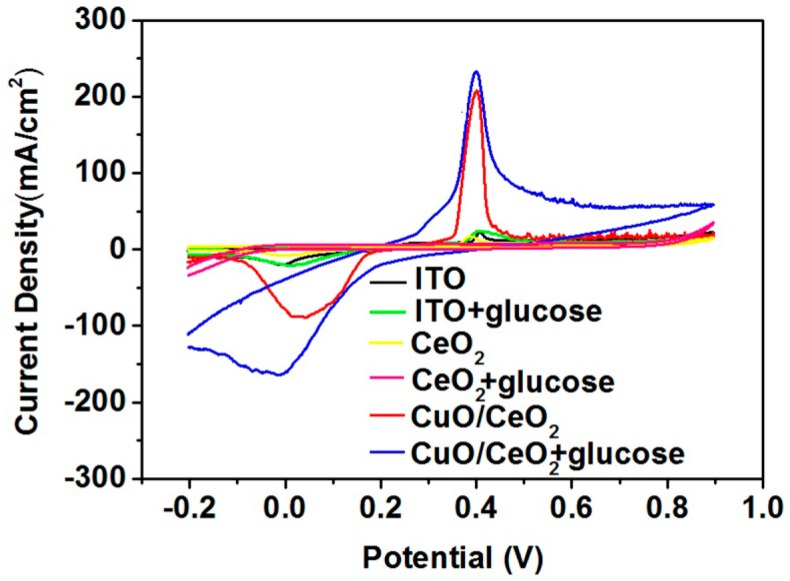
Cyclic voltammograms (CVs) of ITO, CeO_2_/ITO, and CuO/CeO_2_/ITO electrodes in the absence and presence of 5 mM glucose in 0.1 M phosphate buffer (PB) solution at 50 mV/s scan rate.

Figure 5 shows the cyclic voltammograms (CVs) of the electrodes by cycling the electrode potential continuously between −0.2 and 0.9 V against the Ag/AgCl reference electrode. The CuO/CeO_2_ nanocomposite significantly promotes glucose oxidation. The oxidation peak at approximately 0.4 V can correspond to the Cu(II)/Cu(III) redox couple [14,15]. The reaction in the blank phosphate buffer (PB) solution can be expressed as Equation (1) [9]. After glucose is added, electrons can be delivered from glucose to the electrode; as a result, the peak current increases. The reaction can be depicted in Equation (2) [9].

CuO + OH^−^ + e^−^ → CuO(OH) (1)

2CuO(OH) + glucose → 2CuO + gluconolactone + H_2_O (2)

Bare indium-tin oxide (ITO) and pure CeO_2_ remain unchanged after 5 mM glucose is added. This finding indicates that both electrodes do not exhibit specific properties that enable a glucose redox reaction. Given that pure CeO_2_ exhibits a lower charge transfer resistance than the CuO/CeO_2_ nanocomposite does (Figure 4), we considered that the role of CeO_2_ in the nanocomposite is mainly to accelerate the electron transfer while the role of CuO is to dominate the redox reaction of glucose.

**Figure 6:**
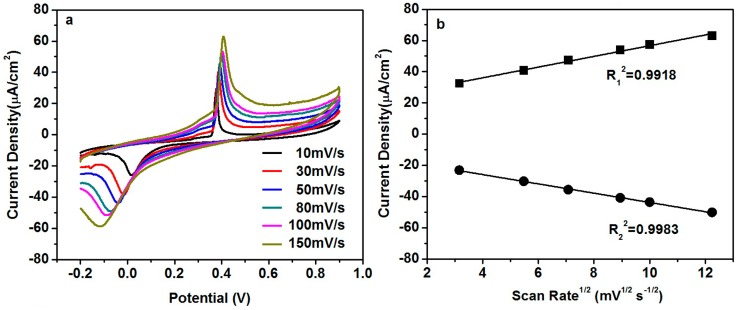
(**a**) CVs of CuO/CeO_2_/ITO electrode in 0.1 M PB solution containing 5 mM glucose at different scan rate; (**b**) Plot of the peak current with square root of the scan rate.

Figure 6a illustrates the influence of the scan rate on the current response. The magnitude of the peak current increases as the scan rate increases. The peak current is quite linear to the square root of the scan rate (Figure 6b). Such linearity indicates the diffusion-controlled kinetics at the electrode surface [16].

**Figure 7:**
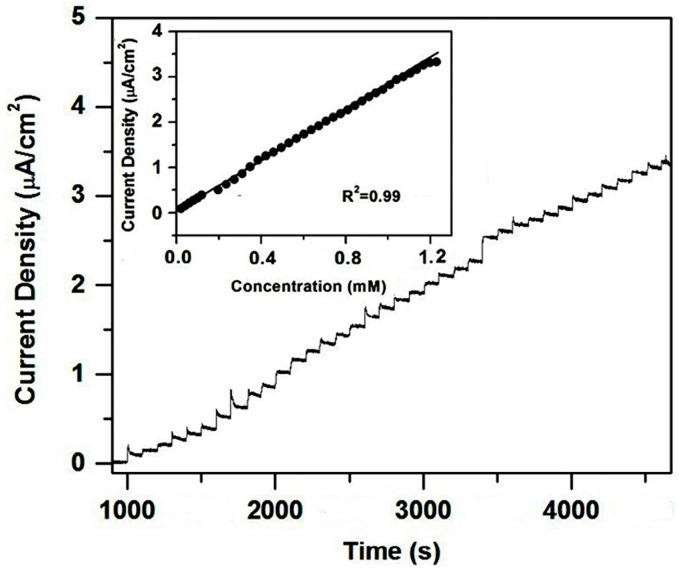
Amperometric response of CuO/CeO_2_/ITO electrode measured at 0.4 V in 0.1 M PB solution with successive addition of 10 mM glucose. Inset is the plot of current response with glucose concentration.

Figure 7 reveals the amperometric response of the CuO/CeO_2_ nanocomposite after 1 mM is successively added to 0.1 M PB solution at 0.4 V. The current increases as glucose is added because of the good catalytic properties of the electrode. The response time is approximately 5~8 s. This amperometric response curve can be used to construct the calibration curve of the electrode (inset of Figure 7). The sensitivity of 2.77 μA mM^−1^cm^−2^ can be obtained from the slope of the calibration curve with a correlation coefficient of 0.99. The limit of detection is 10 μM at a signal-to-noise ratio of 3. The performance of our biosensor is comparable to other CeO_2_-based glucose biosensors which generally contain enzymes or noble metals (Table 1).

**Figure 8:**
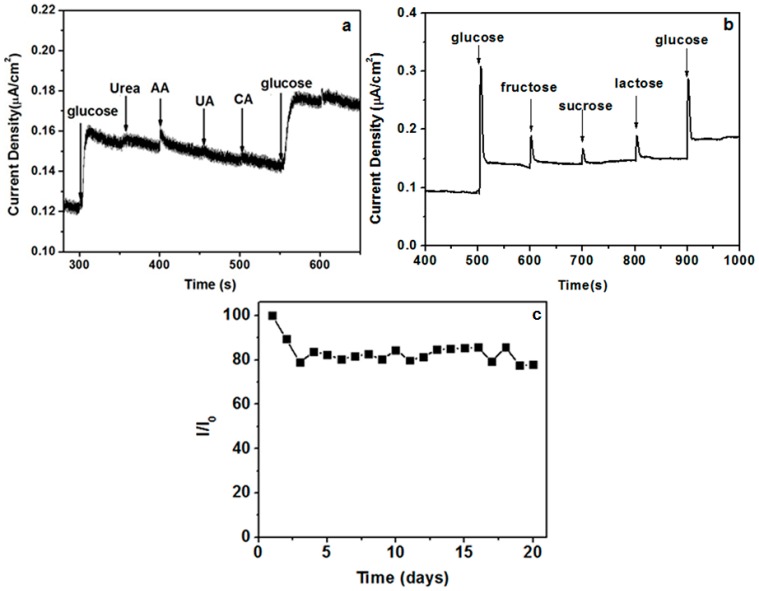
(**a**) Selectivity of CuO/CeO_2_/ITO electrode upon continuous adding 1 mM of glucose, urea, ascorbic acid (AA), uric acid (UA), citric acid (CA), and glucose at 0.4 V in 0.1 M PB solution; (**b**) Selectivity of CuO/CeO_2_/ITO electrode upon continuous adding 1 mM of glucose, fructose, sucrose, lactose, and glucose at 0.4 V in 0.1 M PB solution; (**c**) Stability of CuO/CeO_2_/ITO electrode at ambient conditions for 20 days using 5 mM glucose in 0.1 M PB solution at 0.4 V.

The influence of interfering species on the amperometric response of the CuO/CeO_2_ nanocomposite was investigated through the successive addition of equal concentrations (1 mM) of glucose, ascorbic acid (AA), uric acid (UA), citric acid (CA), and glucose. The sensor exhibits a good anti-interference ability (Figure 8a). Besides, other sugars have little interference toward glucose (Figure 8b). The stability of the CuO/CeO_2_ nanocomposite was examined by measuring its current response (I) daily under ambient conditions for 20 days. In this period, the sensor retained 80% of its original value (I_0_; Figure 8c).

The sensing performance in a real sample was also evaluated. Glucose was added into fruit juice to make a mixed standard solution, and the amperometric detection was carried out at 0.4 V in 0.1 M PB solution after injecting this mixed standard solution. The sensor demonstrated a fairly good performance in recovery (Table 2).

## 3. Materials and Methods 

Most chemicals, such as cerium nitrate, copper sulfate, disodium hydrogen phosphate, sodium dihydrogen phosphate, and glucose, were purchased from Sigma-Aldrich (St. Louis, MO, USA) and used directly without any purification. Other chemicals, including ammonia, urea, ascorbic acid (AA), uric acid (UA), and citric acid (CA) were obtained from Aladdin Chemicals (Shanghai, China), and used as received.

CeO_2_ was synthesized through non-isothermal precipitation. Ammonia was slowly added to 100 mL of 0.20 M cerium nitrate aqueous solution at 70 °C. Yellow precipitates were formed immediately. After 5 min, the reaction mixture was transferred into a water bath, in which the reaction continued for 24 h at 0 °C. Finally, the precipitate was filtered, washed with a large amount of ethanol and de-ionized water to remove the residue, and dried at 60 °C for further application.

CuO/CeO_2_ nanocomposites were prepared via wet impregnation. In brief, CeO_2_ (0.1722 g) was immersed in 0.005 M aqueous copper sulfate solution for 10 h. Subsequently, impregnated CeO_2_ was filtered and calcined at 650 °C in air for 4 h to produce the CuO/CeO_2_ nanocomposites.

Transmission electron microscopy (TEM) was performed with a JEM2100 instrument (JEOL, Tokyo, Japan). X-ray photoelectron spectroscopy (XPS) measurements were conducted by using an Amicus Budget spectrometer (Shimadzu, Tokyo, Japan) with a Mg Kα X-ray source (1253.6 eV) operating at 10 kV and 10 mA. X-ray diffraction (XRD) analysis was carried out with a DX2700 diffractometer (HAOYUAN, Dandong, China) through Cu Kα radiation. 

The CuO/CeO_2_ nanocomposite suspension (5 mg/mL; 100 μL) was casted on the surface of an indium-tin oxide (ITO)-coated glass and dried at 60 °C to form a nanocomposite film. This process was also employed to fabricate a pure CeO_2_ film for comparison. The effective area of electrode was set at 0.5 cm^2^, which was established by covering the unnecessary area with epoxy.

Electrochemical measurements were conducted using a CHI630D electrochemical analyzer (CHENHUA, Shanghai, China). All of the experiments were performed in 0.1 M phosphate buffer (PB) solution (pH = 7.4) with a three-electrode electrochemical cell by using an ITO-based working electrode, a glassy carbon counter electrode, and an Ag/AgCl reference electrode.

## 4. Conclusions 

This study developed a non-enzymatic glucose biosensor based on the CuO/CeO_2_ nanocomposite-modified ITO. The good performance is related to the synergetic effects of the two components. The CuO component dominated the redox reaction of the glucose. The CeO_2_ component accelerated the electron transfer of the electrode. The CuO/CeO_2_ nanocomposite biosensor yields a reasonably good sensitivity of 2.77 μA mM^−1^cm^−2^ and an estimated detection limit of 10 μM. The biosensor is stable and selective.

## Figures and Tables

**Table 1:** Comparison of performance obtained from different cerium oxide (CeO_2_)-based glucose biosensors.

Working Electrode	Detection Limit (μM)	Sensitivity (μA mM^−1^cm^−2^)	References
GOx ^a^/CeO_2_/Au	12.0	0.051	[17]
GOx/CeO_2_/ITO	100	0.165	[8]
GOx/CeO_2_/graphene/FTO ^b^	2	7.198	[18]
CeO_2_/Au/CPE ^c^	10	57.5	[19]
CeO_2_/Au	10	44	[20]
CuO/CeO_2_/ITO	10	2.77	This work

^a^ glucose oxidase; ^b^ fluorine-doped tin oxide; ^c^ carbon paste electrode.

**Table 2:** The results of detecting glucose concentration in fruit juice.

Spiked (μM)	Found (μM)	Recovery (%)
0	50.7	-
25	74.3	94.4
50	98.7	96.0
75	122.3	95.5
100	153.3	102.6
